# Rapidly Progressive Glomerulonephritis Secondary to IgA Nephropathy in a Patient with Systemic Lupus Erythematosus

**DOI:** 10.1155/2019/8354823

**Published:** 2019-03-05

**Authors:** Amol M. Patel, Lily Anne Romero Karam, Stephanie C. Fuentes Rojas, Warren E. Redfearn, Luan D. Truong, Juan M. Gonzalez

**Affiliations:** ^1^Department of Internal Medicine, Houston Methodist Hospital, Houston, TX, USA; ^2^Department of Pathology and Genomic Medicine, Houston Methodist Hospital, Houston, TX, USA; ^3^Department of Internal Medicine, Division of Nephrology, Houston, TX, USA

## Abstract

Lupus nephritis is a common manifestation of systemic lupus erythematosus (SLE). IgA nephropathy is a common type of primary glomerulonephritis. Renal manifestations in SLE patients are often due to lupus nephritis; however, renal diseases unrelated to lupus nephritis are rarely reported. While crescentic IgA nephropathy with rapid clinical progression is rare, its development in patients with SLE in the absence of lupus nephritis is even more unusual. A 74-year-old woman with a history of SLE without known renal involvement, chronic kidney disease stage IIIa, congestive heart failure, hypertension, and type 2 diabetes mellitus presented with acute kidney injury. Her creatinine continued to rise rapidly. Renal biopsy revealed mesangial proliferative glomerulonephritis with crescent formation. Immunofluorescent staining showed IgA and C3 mesangial deposition and absence of C4 and C1q, consistent with IgA nephropathy. She received a course of methylprednisolone and plasmapheresis. Unfortunately, her renal function continued to deteriorate, and she was started on hemodialysis which was continued after hospital discharge. This case illustrates crescentic IgA nephropathy without lupus nephritis as the cause of acute kidney injury in a patient with SLE. It highlights the observation that renal diseases other than lupus nephritis can develop in SLE patients.

## 1. Introduction

Renal disease is common among patients with systemic lupus erythematosus (SLE); however, renal diseases unrelated to lupus nephritis (LN) in these patients are rarely reported.

LN affects about 50% of patients with SLE [1]. Clinically, LN is divided into six subtypes: minimal mesangial LN (class I), mesangial proliferative LN (class II), focal proliferative LN (class III), diffuse proliferative LN (class IV), membranous LN (class V), and advanced sclerosing LN (class VI) [2]. On histology, LN is classically characterized by the so-called “full house” pattern under immunofluorescent microscopy, staining positively for IgG, IgA, IgM, C3, and C1q [3].

Globally, the most commonly observed glomerulopathy is IgA nephropathy (IgAN) [4]. It is characterized by circulating IgA immune complexes which contribute to glomerular inflammation, glomerular capillary damage, and mesangial proliferation. The range of clinical manifestations of IgAN is broad, from asymptomatic microscopic hematuria to rapidly progressive glomerulonephritis (RPGN). Although the two most common clinical presentations are asymptomatic hematuria and progressive kidney disease, IgAN can be fatal with the largest case series of 113 patients showing a rate of progression to ESRD of 42.5% at 1 year despite immunotherapy [5].

We report a case of acute renal injury secondary to RPGN of IgAN in a patient with SLE in the absence of LN. RPGN of IgAN is rare, and more unusual is this presentation in a patient with SLE without LN.

## 2. Case Presentation

A 74-year-old woman with a history of SLE on hydroxychloroquine without known renal involvement, chronic kidney disease stage (CKD) IIIa, heart failure with reduced ejection fraction, hypertension, and type 2 diabetes mellitus (T2DM) presented to the emergency department with progressive chest “heaviness” which had started several days prior to admission. In addition, she complained of swelling in both feet and at least three-pound weight gain.

Upon admission she was afebrile, blood pressure ranged 150-180/70-80 mm Hg, heart rate was about 50 bpm, and oxygen saturation was above 95% on room air. She was a well-developed, nonobese woman in no significant distress and nontachypneic. Pertinent physical exam findings included no jugular venous distention, no crackles audible at base of lungs, heart with regular rate and rhythm with no extra sounds or murmurs, nondistended abdomen, trace peripheral leg edema, and no visible rashes. Laboratory tests were significant for sodium 129 mEq/L, potassium 4.2 mEq/L, chloride 98 mEq/L, CO2 20 mEq/L, BUN 46 mg/dL, and creatinine 3.4 mg/dL. Her baseline creatinine based on the most recent reading one month prior to admission was 1.1 mg/dL. Urinalysis was significant for a specific gravity of 1.006, RBC 142, and WBC 8 per HPF. No casts of dysmorphic cells were seen. Proteinuria was 2+, and a random urine protein-to-creatinine ratio was 1.48.

**Figure 1:**
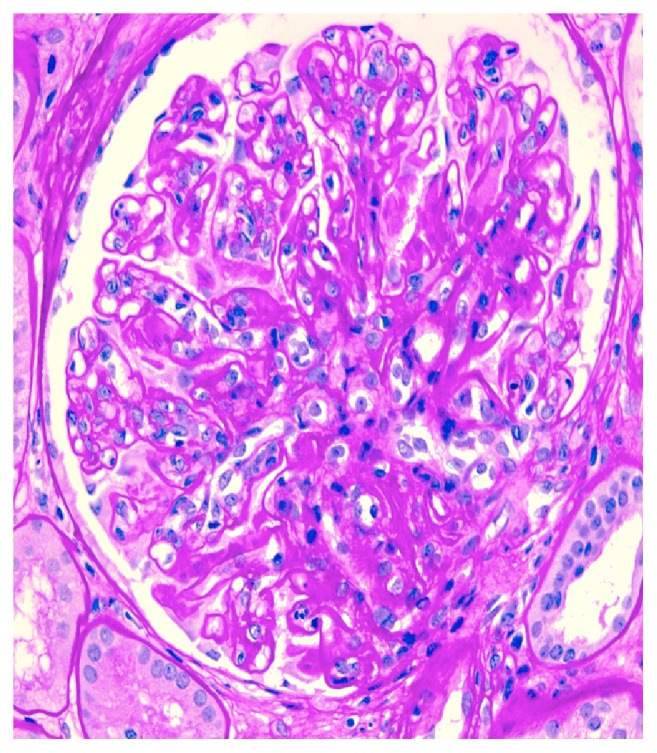
There is global moderate mesangial siderosis and hypercellularity (periodic acid-Schiff, x400).

**Figure 2:**
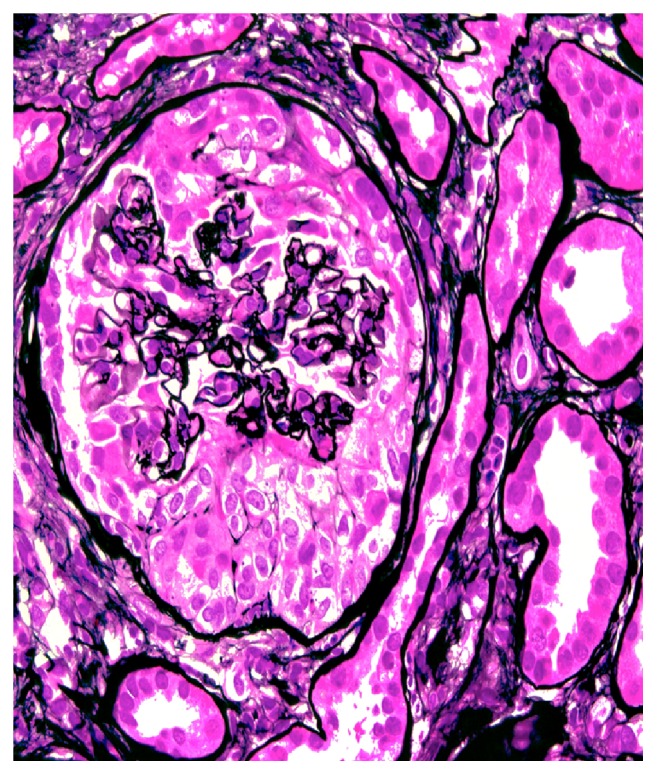
Circumferential cellular crescent is present, associated with collapse of the underlying glomerular capillaries (methenamine silver, x400).

**Figure 3:**
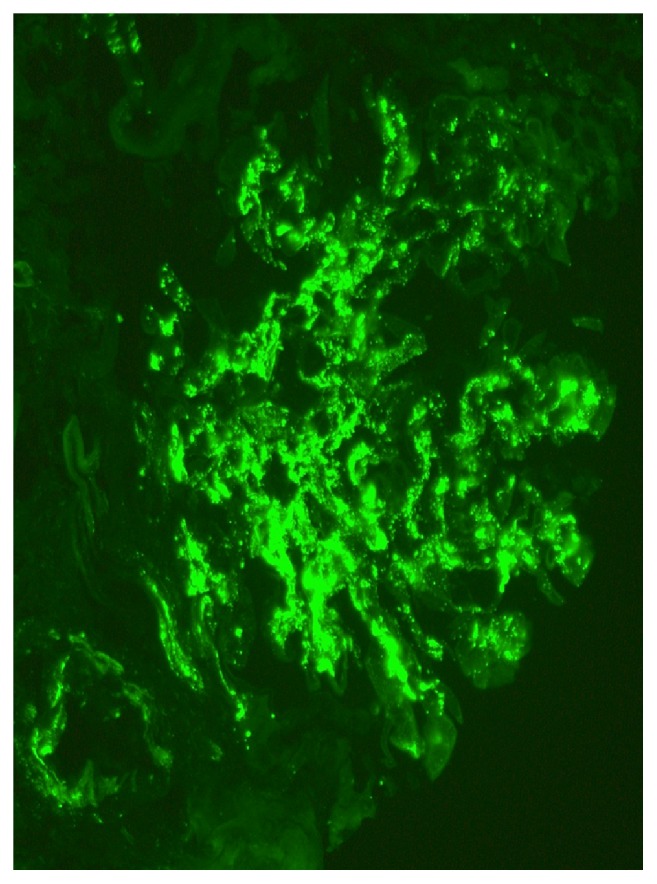
Global 3+ mesangial deposit of immunoglobulin A is present (immunofluorescent, x 400).

**Figure 4:**
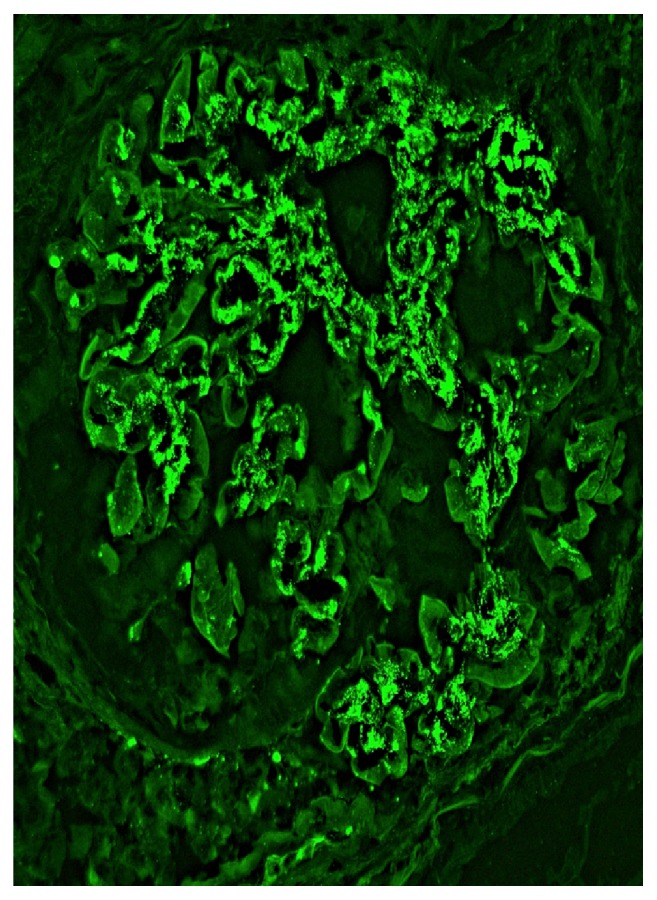
Global 3+ mesangial deposit of complement component C3 is present (immunofluorescent, x 400).

**Figure 5:**
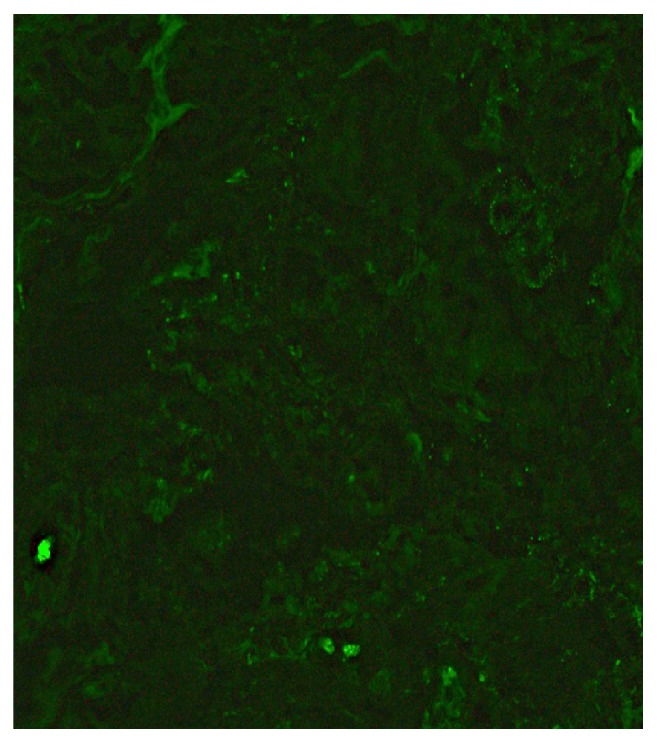
There is no glomerular deposition of complement component C1q present (immunofluorescent, x 400).

**Figure 6:**
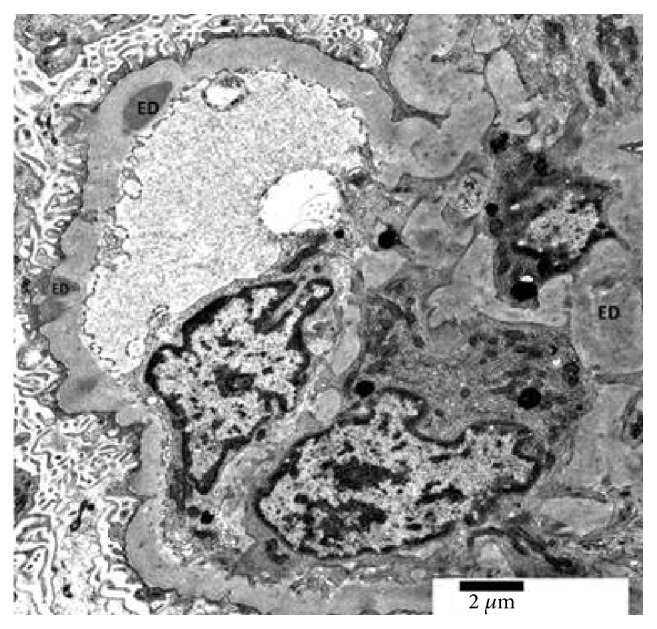
There is mesangial hypercellularity and mild thickening of glomerular basement membrane along with electron dense deposits (ED) in mesangial, subendothelial, and subepithelial locations (electron microscopy, x 8000).

**Figure 7:**
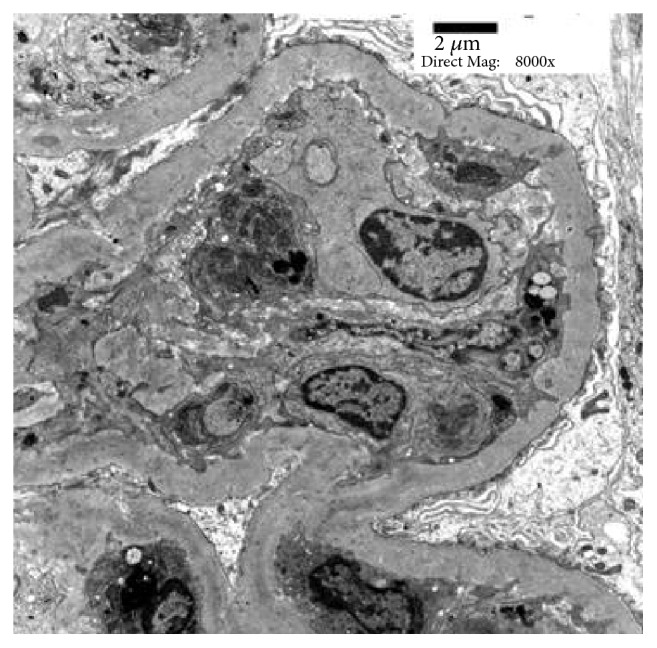
There are endocapillary cell hypercellularity and several small electron dense deposits within the glomerular basement membrane (electron microscopy, x 8000).

Her creatinine continued to rise rapidly, and by day 6 of hospitalization it was 6.4 mg/dL. Due to these findings, a renal biopsy was performed. Up to 21 glomeruli were present per tissue section, and two of them showed global sclerosis. Two other glomeruli showed segmental sclerosis and the remaining glomeruli were open. There was global diffuse marked mesangial sclerosis and hypercellularity, and there was no obvious endocapillary cell proliferation or inflammatory cell infiltrate. Three of the open glomeruli also displayed segmental or circumferential predominantly cellular crescent. There was multifocal chronic tubulointerstitial injury characterized by atrophic tubules, interstitial fibrosis, and mild mononuclear inflammatory cell infiltrate, all which accounted for about 20-40% of the cortical tissue area. Figures 1 and 2 are biopsies seen in light microscopy with the former showing mesangial proliferation and the latter demonstrating a crescent on silver stain. Immunofluorescent staining showed marked IgA and C3 mesangial deposition, weak linear IgG staining of the glomerular basement membrane, and absence of C4 and C1q which was consistent with IgA nephropathy. There are also changes suggestive of diabetic nephropathy, in keeping with the clinical history of diabetes. These changes include thickened lamina densa and linear IgG of the glomerular basement membrane and mesangial sclerosis. Immunofluorescent staining positive for IgA and C3 is seen in Figures 3 and 4, respectively. Staining for C4 and C1q was both negative with the latter seen on Figure 5. In addition, electron microscopy showed uniform thickening of lamina densa of glomerular basement membrane, marked mesangial sclerosis, and hypercellularity, and some electron dense deposits were identified in the mesangial areas and subepithelial location which can be seen in Figures 6 and 7. Ultimately, given the histological findings, the biopsy was diagnosed as mesangial proliferative glomerulonephritis with crescent formation. Findings were not comparable with lupus nephritis, especially the immunofluorescent findings. The significant mesangial IgA staining raised the likelihood of IgA nephropathy.

Her serologic work-up included anti-dsDNA antibody (1:80), ANA (1:320), and normal serum complement levels. With these biopsy results in the setting of worsening kidney function, she was diagnosed with immune complex RPGN secondary to IgAN.

She subsequently received intravenous methylprednisolone 500 mg daily for three days and five rounds of plasmapheresis. Unfortunately, her renal function continued to deteriorate, and she was started on hemodialysis. She was discharged with instructions to take prednisone 60 mg daily for one month with a taper. Her clinical symptoms showed mild improvement, however, there was no significant recovery in kidney function, and she was declared to have end-stage renal disease (ESRD).

## 3. Discussion

Renal disease is a common complication in patients with SLE and can affect up to 50% of the population. While LN must be kept high on the differential in SLE patients presenting with renal dysfunction, it is important to consider these patients are not exempt from renal diseases which affect the general population. Our patient with SLE had known CKD stage IIIa which had been attributed as a complication of T2DM and hypertension, but she was ultimately found to have concomitant IgAN. In addition, the biopsy-proven RPGN of IgAN was without characteristics classically seen in LN.

As the most common cause of primary glomerulonephritis, IgAN was initially seen to run a benign course. However, it is now recognized almost half of the patients affected progress to ESRD. There are several clinical factors which have been known to be markers for progression and severe disease: elevated serum creatinine, hypertension, and persistent proteinuria above 1000/day [6]. Our patient demonstrated all three of these markers upon diagnosis as she presented with blood pressure ranging from 150 to 180/70-80 with a known history of hypertension. She also had a baseline serum creatinine of 1.1 mg/dL with a calculated eGFR of 44 mL/min/1.73m^2^. In addition, her urine spot protein-creatinine ratio was 1.48 to suggest approximately protein excretion of 1480 mg per day. Other notable risk factors for progression of disease include obesity, hypertriglyceridemia, hyperuricemia, and smoking [7–9]. Our patient was only notable to be a former smoker who had quit smoking in the remote past.

Lastly, specific histological predictors have been shown to be associated with more severe disease. These include mesangial hypercellularity, endocapillary hypercellularity, segmental glomerulosclerosis, tubular atrophy, and crescent formation. Formally, this is known by the Oxford Classification of IgA nephropathy as the MEST score (or MEST-C score if accounting for crescents). The MEST score with clinical data at the time of biopsy has been shown to provide the same predictive power as monitoring clinical data for two years [10]. Based on histopathological results, our patient would be classified as M1, E0, S0, T1, and C1, with each metric having specific prognostic utility.

The patient was also found to have immune complex RPGN which is clinically and histologically correlated with the rapid progressive loss of renal function with crescent formation and biopsy-proven IgAN. Untreated RPGN typically progresses to end-stage renal disease over a period of weeks to a few months. The treatment for RPGN specifically in patients with IgAN has not been studied in randomized clinical trials; however, observational data suggests regimens used like those of idiopathic crescentic glomerulonephritis may provide possible benefit. This includes intravenous pulse methylprednisolone followed by plasmapheresis, oral prednisone, or cyclophosphamide (either oral or intravenous) [11]. Our patient was treated with intravenous methylprednisolone for three days followed by five days of plasmapheresis and continued oral prednisone. Although her clinical symptoms slightly improved, her renal function continued to deteriorate, and she was declared ESRD.

A previous study analyzed 251 SLE patients with ESRD using United States surveillance data. The results of the study showed that 21.1% of patients had ESRD attributed to a cause other than SLE. The majority of this subset was due to hypertension and diabetes. However, only 3.1% of the total study population had ESRD attributed to a non-SLE glomerulonephritis [12], and this is the subset which our patient fits into. Furthermore, she presented with crescentic IgA nephropathy, which although is the most commonly observed glomerulonephritis globally, it rarely presents with RPGN.

To our knowledge there is a large void of studies which report patients with SLE who develop non-LN-related crescentic glomerulonephritis. This is a case of a patient with known SLE and CKD who presented with acute kidney injury ultimately diagnosed with RPGN of IgA nephropathy without lupus nephritis. This highlights the importance renal biopsy to guide diagnosis and treatment in a patient with multiple risk factors for renal disease.
